# Supplementary material for: The genome sequence of the Two-tailed Pasha, Charaxes jasius (Linnaeus, 1767) (Lepidoptera: Nymphalidae)
Source: Wellcome Open Res. 2026 Jan 23;11:61. [Version 1] doi: 10.12688/wellcomeopenres.25789.1 (PMC13003222; doi:10.12688/wellcomeopenres.25789.1)
Supplement: Peer review history

# Reviewer report: Reviewer response for version 1

**DOI:** 10.21956/wellcomeopenres.28390.r149600

**Published:** 2026-03-19

**Authors:** Yoshiaki Morino

**Affiliations:** 1 University of Tsukuba, Tsukuba, Japan

This manuscript presents a high-quality, chromosome-level, haplotype-resolved genome assembly for *Charaxes jasius*. The reported assembly metrics are strong and support the authors’ conclusion that this is a reference-quality resource. In particular, haplotype 1 has a total span of 465.23 Mb, with 99.91% of the assembly assigned to 26 chromosomal pseudomolecules, including the W and Z sex chromosomes. The scaffold N50 is 16.32 Mb, indicating a highly contiguous and well-resolved assembly.

 The quality assessment is also convincing. BUSCO completeness is 98.7% using the *lepidoptera_odb10* dataset, with 98.4% single-copy and only 0.3% duplicated BUSCOs, which is consistent with a highly complete assembly. The consensus quality is likewise very high, with a reported QV of 62.5 for haplotype 1 and 62.6 for the combined haplotypes, meeting the recommended reference standard.

 The sequencing and assembly strategy is appropriate and clearly described. The genome was generated from 15.76 Gb of PacBio HiFi data, corresponding to approximately 35× coverage based on the estimated haploid genome size, and scaffolded with 96.9 Gb of Hi-C data. The use of HiFi reads, Hi-C phasing, manual curation, and standard quality-control tools is fully in line with current best practices.

 Overall, I found the manuscript technically sound, clearly written, and suitable for Indexing in its present form. The assembly statistics, quality metrics, and data presentation are all of a high standard, and I have no major concerns regarding the methodology or the utility of the dataset. This genome should represent a valuable resource for future studies of butterfly comparative genomics, chromosome evolution, and the evolutionary biology of *Charaxes* and related nymphalid lineages.

Are sufficient details of methods and materials provided to allow replication by others?

Yes

Is the rationale for creating the dataset(s) clearly described?

Yes

Are the datasets clearly presented in a useable and accessible format?

Yes

Are the protocols appropriate and is the work technically sound?

Yes

Reviewer Expertise:

marine invertebrate, Evo-Devo

I confirm that I have read this submission and believe that I have an appropriate level of expertise to confirm that it is of an acceptable scientific standard.

# Reviewer report: Reviewer response for version 1

**DOI:** 10.21956/wellcomeopenres.28390.r146709

**Published:** 2026-02-20

**Authors:** James B Whitfield

**Affiliations:** 1 University of Illinois at Urbana-Champaign, Champaign, USA

This paper presents a report on a reference-quality genome obtained for the only member of the mainly tropical nymphalid butterfly subfamily Charaxinae to be found in Europe. The Background section of the paper provides a nice summary of the biology and conservation status of this species.  I was a bit surprised that there was no mention. of the significance various nymphalid butterflies in studying mechanisms of developmental evolution of color patterns in mimicry and aposematism (e .g. Beldade & Brakefield 2002 Nature; Abbasi & Marcus 2015 Evolution & Development; Tian and Monteiro 2026 Curr Opin Insect Science; just to name a very few examples). No doubt having a genome from a distinct nymphalid subfamily might be useful for phylogenetic perspective, especially for the relevant evo-devo genes?

 Otherwise, the paper seems very well prepared and clearly written, and the methods are certainly appropriate and relatively state of the art.  The presentation of genomic details seems rather standard for these reports, and straightforward to interpret.

Are sufficient details of methods and materials provided to allow replication by others?

Yes

Is the rationale for creating the dataset(s) clearly described?

Yes

Are the datasets clearly presented in a useable and accessible format?

Yes

Are the protocols appropriate and is the work technically sound?

Yes

Reviewer Expertise:

Systematics, phylogeny and comparative genomics of parasitoid wasps and their associated mutualistic viruses

I confirm that I have read this submission and believe that I have an appropriate level of expertise to confirm that it is of an acceptable scientific standard.
